# Chronic atrazine exposure increases the expression of genes associated with GABAergic and glutamatergic systems in the brain of male albino rat

**DOI:** 10.3389/ftox.2022.933300

**Published:** 2022-08-22

**Authors:** D. Y. Reyes-Bravo, P. Villalobos-Aguilera, J. T. Almonte-Zepeda, M. S. Mendoza-Trejo, M. Giordano, A. Orozco, V. M. Rodríguez

**Affiliations:** ^1^ Departamento de Neurobiología Conductual y Cognitiva, Instituto de Neurobiología, Universidad Nacional Autónoma de México, Querétaro, México; ^2^ Departamento de Neurobiología Celular y Molecular, Instituto de Neurobiología, Universidad Nacional Autónoma de México, Querétaro, México

**Keywords:** herbicides, GABA, glutamate, albino rat, neurodegenenerative diseases, mRNA expression

## Abstract

The herbicide atrazine (ATR; 2-chloro-4-ethylamino-6-isopropylamino-s-triazine) is widely used to destroy grasses and broadleaf weeds in crops and some fruits. Studies in rodents have shown that acute, repeated or chronic exposure to ATR is associated with alterations in the nigrostriatal dopaminergic pathway, whereas its effects on GABAergic and glutamatergic pathways have only recently been reported. Sprague-Dawley male rats were exposed daily to 1 or 10 mg ATR/kg of BW for 13 months to evaluate the ATR effects on GABAergic and glutamatergic systems. At the end of the ATR treatment, the levels of mRNA of several genes involved in the production, vesiculation, reuptake, and receptors of GABA and Glu in the striatum (STR), nucleus accumbens (NAcc), prefrontal cortex (PFC), ventral midbrain (vMID) and hippocampus (HIPP) were evaluated by absolute qPCR. For the GABAergic genes, increased expression of *GAD67* and *Slc32a1* in STR and/or vMID in rats exposed to 1 and/or 10 mg ATR were detected. With regard to the expression of genes involved in the glutamatergic system, *Slc17a6* and *Grin1* in HIPP of rats exposed to 1 and/or 10 mg ATR, increased as was *Gria1* in STR and PFC in the group exposed to 1 mg ATR. In the same fashion, *Slc1a3* expression and *MGLUR1* increased in STR of rats exposed to 1 and 10 mg ATR groups. The expression of the glutaminases *gls* (variants 1 and 2) was greater in STR, NAcc, HIPP, and PFC of rats exposed to 1 and/or 10 mg ATR. These findings show that the GABAergic and, especially glutamatergic systems are targets of ATR exposure.

## Introduction

Atrazine (ATR; 2-chloro-4-ethylamino-6-isopropylamino-s-triazine) is a selective and systemic organochlorinated herbicide that belongs to the chlorotriazines family. This herbicide is a pre-and post-emergent agent used to kill weeds in corn, wheat, sugar cane cultures, among others (Rowe et al., 2006). The widespread use of ATR in several countries worldwide is associated with high human exposure to tainted waters, soils, and food (Jablonowski et al., 2011; Sun et al., 2017).

Studies in rodents have shown that exposure to ATR is associated with alterations in the basal ganglia nuclei’s integrity. The basal ganglia include the striatum, globus pallidus, substantia nigra, and the subthalamic nucleus. These nuclei’s functionality is regulated by the interaction between the cortex, thalamus, and other brain structures by GABAergic, glutamatergic and dopaminergic systems. The basal ganglia nuclei that have GABAergic projection neurons are striatum, entopeduncular nucleus (EP) or internal segment of the globus pallidus in primates (Gpi), substantia nigra pars reticulata (SNpr) (located in the ventral midbrain), and the external segment of the globus pallidus in primates (GPe) (Bolam et al., 2000). It is important to note that the cortex innervates the striatum by a glutamatergic projection; another structure leading to a glutamatergic projection is the subthalamic nucleus that sends its projections to the output nuclei (GPi/SNpr) (Bolam et al., 2000). Furthermore, the substantia nigra pars compacta and ventral tegmental area send their dopaminergic projections to the striatum and nucleus accumbens, respectively (Cooper et al., 2003). It is important to note that these nuclei regulate movement, reward, cognition, memory, and other brain functions.

Several studies have described the effects of ATR exposure on the basal ganglia, the nigrostriatal dopaminergic system being the most studied under several paradigms of treatment such as acute, repeated, or chronic administration. Regarding chronic ATR exposure, daily exposure to 10 mg ATR/kg for 1 year produces hyperactivity, decreases striatal dopamine levels, and diminishes the number of tyrosine hydroxylase positive cells in the substantia nigra pars compacta (Bardullas et al., 2011; Bardullas et al., 2013). The repeated exposure to 100 mg ATR/kg (six IP injections over 2 weeks) causes hypoactivity, decreases in the in striatal specific binding to D1-DA receptors, decreases the striatal levels of dopamine, and produces alterations in the mRNA expression of vesicular monoamine transporter-2 (VMAT-2), dopamine transporter (DAT) and tyrosine hydroxylase (TH) (Rodriguez et al., 2013; Marquez-Ramos et al., 2017) of the male albino rat. While Coban and Filipov (2007) reported decreases in striatal dopamine levels accompanied by a reduced number of tyrosine hydroxylase positive cells in the substantia nigra pars compacta after daily injection of 5—250 mg ATR/kg during 14 days in the C57BL6J male mice.

However, few studies have evaluated the toxic effects of ATR exposure in the GABAergic or glutamatergic neurotransmitter systems in rodents. In this respect, Shafer et al. (1999) found that ATR can interfere with the binding of Ro15-4513 (an agonist of the benzodiazepine site) GABA-A receptors in a non-competitive manner in brain cortical tissue of rats. While Rodriguez et al. (2017) found that the acute exposure to 100 mg/kg ATR caused hypoactivity, which was relatively annulled by saclofen (GABA-B antagonist), and increased the number of c-Fos-positive cells in the central amygdala, substantia nigra, subthalamic nucleus, superior colliculus, and thalamus 90 min after the IP injection. This protocol of ATR exposure did not affect GABA or glutamate tissue levels on the striatum, nucleus accumbens, prefrontal cortex, ventral midbrain, dorsal midbrain, amygdala, or hippocampus. A recent study by Chavez-Pichardo et al. (2020) found that rats chronically treated with 1 or 10 mg ATR/kg for 13 months showed hyperactivity, anxiety, and alterations in tissue levels of GABA, glutamate, and glutamine in striatum, nucleus accumbens, ventral midbrain, the amygdala, and the prefrontal cortex. In addition, they also found increased striatal extracellular levels and increased release of glutamate in rats exposed to 10 mg ATR/kg.

This study is a follow-up study of our previous reports with the specific aim to explore the possible toxic effects of the chronic exposure to 1 or 10 mg ATR/kg on the mRNA expression of genes associated with neurotransmitter production upon GABAergic and glutamatergic signaling pathways, as indicators of possible disarrays in the regulation of various genes that could impact cellular functions or neurological pathways in the striatum, nucleus accumbens, prefrontal cortex, ventral midbrain and hippocampus, regions already reported to be affected by ATR exposure in a similar protocol of treatment (Chavez-Pichardo et al., 2020).

## Materials and methods

### Chemicals

Atrazine was obtained from ChemService (West Chester, PA, United States). Unless otherwise stated, reagents for qPCR were obtained from Sigma-Aldrich (St. Louis, MO, United States).

### Animals

Thirty twenty-one-day-old male Sprague-Dawley rats were obtained from Envigo, Mexico (Mexico City, Mexico). Rats were kept under a 12-h inverted light/dark cycle (lights on at 18:00 h) with access to food and water *ad libitum*.

### Ethics

Experiments were approved by the local Committee of Bioethics and carried outfitting to the Official Mexican Standard NOM- 062-ZOO-1999 titled “Especificaciones técnicas para la producción, cuidado y uso de los animales de laboratorio,” which agrees with the guidelines of the Institutional Animal Care and Use Committee Guidebook (NIH Publication 80–23, Bethesda, MD, United States, 1996).

### Experimental design

**FIGURE 1:**
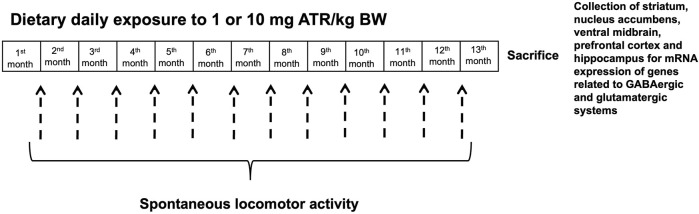
Experimental design.

Male rats were randomly divided into three groups at arrival to the laboratory facilities. Two groups (n = 10 rats per group) were daily exposed to 1 or 10 mg ATR/kg of B.W. mixed with regular Lab Diet in hand-made pellets, while the control group received pellets without ATR for 13 months. Once rats reached 300 g, they were kept at this bodyweight by food restriction, as already reported (Chavez-Pichardo et al., 2020). Locomotor activity (24 h) was evaluated monthly for 1 year. After ending ATR treatment, rats were sacrificed by decapitation, the brain was extracted, and the striatum, nucleus accumbens, prefrontal cortex, ventral midbrain, and hippocampus were frozen −80°C until analysis. Tissue was collected to evaluate mRNA expression of genes related to production, vesiculation, reuptake, and receptors of the GABAergic and the glutamatergic systems (Figure 1).

### Spontaneous locomotor activity

Rats were individually placed in a locomotor activity chamber where spontaneous locomotor activity was measured, and data was collected for 24-h as previously described by our group (Chavez-Pichardo et al., 2020). The parameters recorded to assess the effects of ATR exposure were horizontal activity described as the total number of beam interruptions on the horizontal axis; vertical episode activity, identified as the total number of rearings recorded on the vertical sensor; and the location parameters margin and center distance, defined as the distance traveled in the margin or the center of the activity chamber during a given period.

### Quantitative PCR

Total RNA was extracted from the prefrontal cortex, striatum, nucleus accumbens, hippocampus, and ventral midbrain with Trizol Reagent (Life Technologies) and cDNA was reverse transcribed with Thermo Scientific RevertAid First Strand cDNA Synthesis (Thermo Scientific) from 2 μg of total RNA using 0.5 μg oligo (dT) primer (Thermo Scientific, Wilmington, DE, United States). Specific oligonucleotides were verified with the Real-time qPCR tool IDT. PCR products were obtained using a proofreading DNA polymerase (Thermo Scientific), and they were cloned into pJET1.2/blunt vector (Thermo Scientific). Constructs were verified by sequencing, and standard curves were prepared in the range of 10^5^ to 10^9^ molecules/μL. In all cases, qPCR reactions contained 1 μL of reverse-transcribed product, Maxima SYBR Green/ROX qPCR Master Mix (Thermo Scientific), and 200–500 nm forward and reversed oligonucleotides in a final volume of 10 μL. According to the manufacturer’s instructions, a Step One (Applied Biosystems) instrument was used for detection and data analysis. The absolute mRNA concentration was expressed as molecules per microgram of total mRNA used in the RT reaction, and Ct values were interpolation in the standard curve and normalized using reference gene cyclophilin A in each experimental sample. The list of primers utilized for qPCR are shown in Table 1, and experimental conditions are shown in Table 2.

**TABLE 1:** Primer pairs used in the quantitative PCR reactions.

Gene name (common name)	Symbol	GeneBank	Primer (5´-3´)	Length (bp)	Primer concentration (nM)	Ta (°C)	Reference
Glutaminase transcript variant 1	*Gls*	NM_012569.2	F: CAG​AAC​AGC​CCT​GCA​TGT​TGC​TGC​TG	100	250	61	Cardona et al. (2015)
			R: CCA​CCT​GTC​CTT​GGG​GAA​GGG​GT				
Glutaminase transcript variant 2	*Gls2*	NM_001109968.3	F: GGC​ATT​CCT​TTG​GAC​CAT​TGG​AC	143	250	61	Cardona et al. (2015)
			R: CCT​CTC​CCC​CAG​ACT​TTC​CAT​TC				
Solute carrier family 17 member 6 (Vesicular glutamate transporter)	*Slc17a6*	NM_053427.1	F: AGG​AGC​AAG​CAA​ATT​CTT​TC	106	400	61	Lau et al. (2013)
			R: AGG​AGC​AAG​CAA​ATT​CTT​TC				
Glutamate ionotropic receptor NMDA type subunit 1	*Grin1*	NM_001270602.1	F: ATGGCTTCTGCATAGACC	110	600	61	Lau et al. (2013)
			R: GTTGTTTACCCGCTCCTG				
Glutamate ionotropic receptor kainate type subunit 1	*Grik1*	NM_001111117.1	F: TGC​TAA​ATA​GTT​TCT​GGT​TTG​G	94	400	57	Lau et al. (2013)
			R: ATT​CCT​CCA​ACT​ATT​CTG​GTC				
Glutamate ionotropic receptor AMPA type subunit 1	*Gria1*	M36419.1	F: GAC​TCT​GGC​TCC​ACT​AAA​GA	105	200	61	Lau et al. (2013)
			R: AGT​CCT​CAC​AAA​CAC​AGA​GG				
Solute carrier family 1 member 3 (Excitatory amino acid transporter)	*Slc1a3*	S59158.1	F: GGA​TGA​CAT​CAC​ACT​CAT​CA	108	400	61	Lau et al. (2013) (Forward) Designed by us (Reverse)
			R: GAC​AAG​TGT​TCA​ACA​ATC​CC				
Metabotropic glutamate receptor	*MGLUR1*	M61099.1	F: AGG​AGG​TGT​GGT​TCG​ATG​AG	77	250	57	Merrill et al. (2012)
			R: ATT​AGC​TTC​TGT​GTA​CTG​CAG​ATT​C				
Glutamic acid decarboxylase	*GAD65*	M72422.1	F: AGT​GCC​ACA​GCT​GGA​ACC​A	155	250	57	Merrill et al. (2012)
			R: ACA​CCG​TTC​AGC​TTC​CAC​TTG​T				
Glutamic acid decarboxylase	*GAD67*	M76177.1	F: CAT​CCT​GGT​CAA​GGA​AAA​GG	78	250	57	Merrill et al. (2012)
			R: TGC​TTG​TCT​GGC​TGG​AAG​AG				
Gamma.aminobutyric acid type A receptor subunit alpha 2	*Gabra2*	NM_001135779.2	F: GAC​AAT​GAC​CAC​ATT​AAG​CAT​CAG	356	200	61	Designed by us
			R: TCT​TGG​CTT​CGG​CTG​GCT​TGT​TCT​C				
Solute carrier family 6 member 1 (GABA trasnsporter)	*Slc6a1*	NM_024371.2	F: GCG​CAA​CAT​GCA​CCA​AAT​GAC​A	140	300	61	Wearne et al. (2016)
			R: AGA​CCA​CCT​TTC​CAG​TCC​ATC​CAA				
Solute carrier family 32 member 1 (Vesicular GABA transporter)	*Slc32a1*	NM_031782.2	F: AAA​CGC​CAT​TCA​GGG​CAT​GTT​C	197	300	61	Wearne et al. (2016)
			R: CGT​TAG​CTA​TGG​CCA​CAT​ACG​A				
Peptidylprolyl isomerase A (Cyclophilin A)	Ppia	NM_017101.1	F: GGG​TTC​CTC​CTT​TCA​CAG​A	320	500	61	Designed by us
			R: GGC​AAG​ACC​AGC​AAG​AAG​A				

**TABLE 2:** Experimental conditions for quantitative PCR.

Gene name (common name)	Symbol	Length (bp)	PCR cycles conditions			
			Denaturation time and temperature	Alignment time and temperature	Extension time and temperature	Number of cycles
Glutaminase transcript variant 1	*Gls*	100	10´´ at 95°C	10´´ at 61°C	8´´ at 72°C	40
Glutaminase transcript variant 2	*Gls2*	143	10´´ at 95°C	10´´at 61°C	10´´ at 72°C	40
Solute carrier family 17 member 6 (Vesicular glutamate transporter)	*Slc17a6*	106	10´´ at 95°C	10´´ at 61°C	8´´ at 72°C	40
Glutamate ionotropic receptor NMDA type subunit 1	*Grin1*	110	10´´ at 95°C	10´´ at 61°C	8´´ at 72°C	40
Glutamate ionotropic receptor kainate type subunit 1	*Grik1*	94	10´´ at 95°C	10´´ at 57°C	8´´ at 72°C	50
Glutamate ionotropic receptor AMPA type subunit 1	*Gria1*	105	10´´ at 95°C	10´´ at 61°C	8´´ at 72°C	40
Solute carrier family 1 member 3 (Excitatory amino acid transporter)	*Slc1a3*	108	10´´ at 95°C	10´´ at 61°C	8´´ at 72°C	40
Metabotropic glutamate receptor	*MGLUR1*	77	10´´ at 95°C	10´´ at 57°C	8´´ at 72°C	40
Glutamic acid decarboxylase	*GAD65*	155	10´´ at 95°C	10´´ at 57°C	10´´ at 72°C	40
Glutamic acid decarboxylase	*GAD67*	78	10´´ at 95°C	10´´ at 57°C	8´´ at 72°C	40
Gamma.aminobutyric acid type A receptor subunit alpha 2	*Gabra2*	356	1´ at 95°C	1´ at 61°C	1´ at 72°C	40
Solute carrier family 6 member 1 (GABA trasnsporter)	*Slc6a1*	140	10´´ at 95°C	10´´ at 61°C	10´´ at 72°C	40
Solute carrier family 32 member 1 (Glutamate vesicular transporter)	*Slc32a1*	197	10´´ at 95°C	10´´ at 61°C	20´´ at 72°C	40
Peptidylprolyl isomerase A (Cyclophilin A)	Ppia	320	10´´ at 95°C	10´´ at 61°C	10´´ at 72°C	40

### Statistical analysis

Spontaneous locomotor activity was analyzed using a two-way analysis of variance with repeated measures (RMANOVA; treatment x time) followed by post hoc tests (Fisher’s LSD). The mRNA statistical analysis was evaluated with nonparametric tests (Kruskal-Wallis and Mann-Whitney’s U tests for pairwise comparisons) because the data did not meet the requirements for parametric tests. Statistical significance level *p* < 0.05 was defined in all cases.

## Results

### Chronic ATR exposure causes alterations in spontaneous locomotor activity after one year of sustained treatment

**FIGURE 2:**
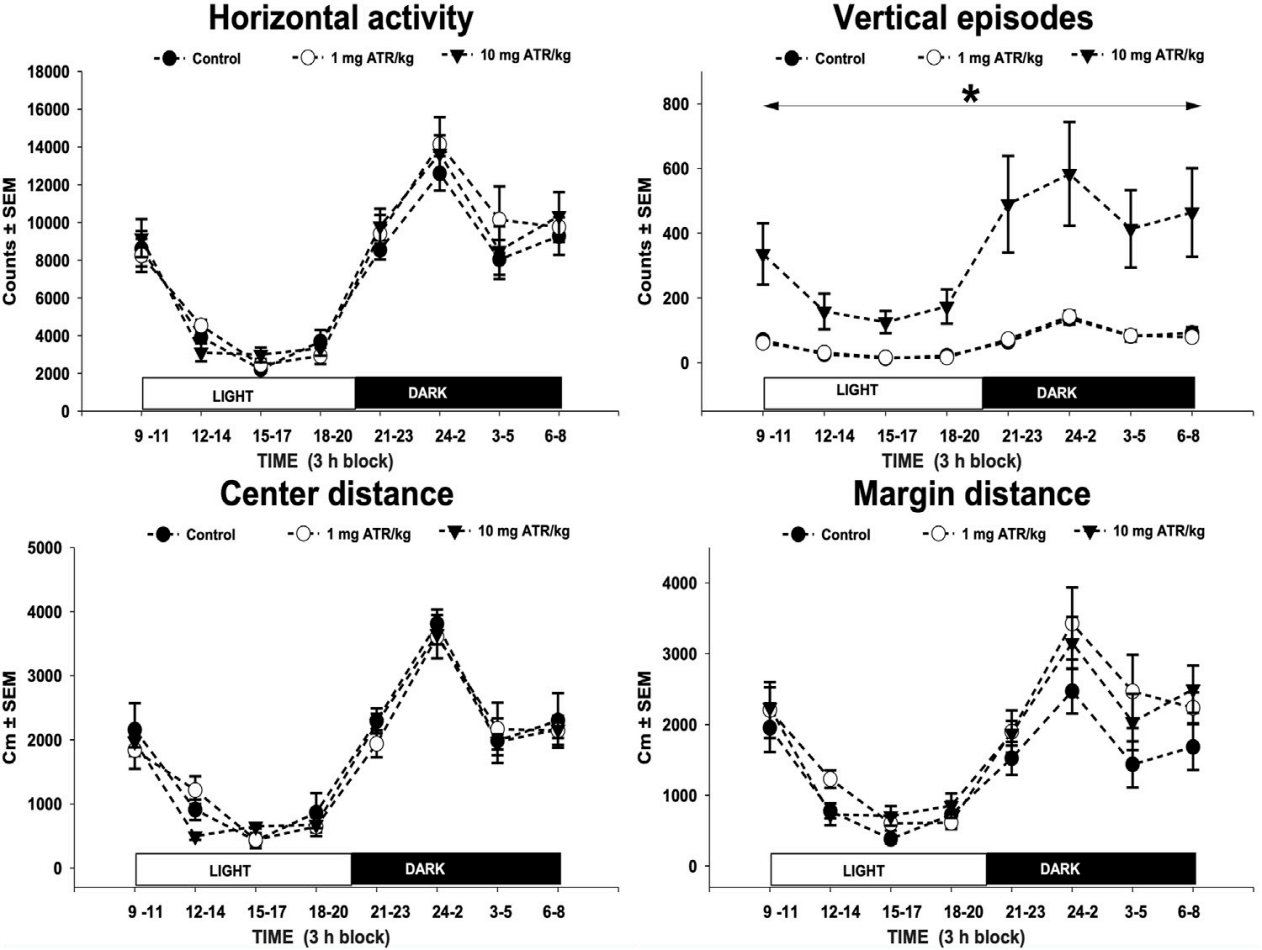
Horizontal activity, vertical episode activity, center distance, and margin distance of recorded throughout a 24-h dark/light cycle. Spontaneous locomotor activity was evaluated at 12 months of exposure to 1 or 10 mg ATR/kg. Chronic exposure to 10 mg ATR/kg caused a significant increase in vertical activity in comparison to 1 mg ATR/kg and control groups. Data were analyzed using a two-way analysis of variance with repeated measures (treatment x time) followed by Fisher’s LSD tests for pairwise comparisons.* Denotes differences of 10 mg ATR/kg group in comparison to the 1 mg ATR/kg and control groups, *p* < 0.05 (*n* = 10)*.*

Only after 1 year of daily ATR treatment there were found significant treatment effects [F = (2, 27) = 8.392, *p* = 0.0015] and 24-h sampling effects [F = (7, 189) = 18.768, *p* < 0.0001] and interaction effects (ATR treatment X sample) [F = (7, 189) = 5.294, *p* < 0.0001] for vertical episode activity. The one-way ANOVAs unveiled differences among groups along the 24-h-long recording [F´s = (2, 27) = 6.066–8.314, *p* < 0.05]. The post hoc tests showed hyperactivity during the 24-h recording in the group treated with 10 mg ATR/kg in comparison to 1 mg ATR/kg and control groups (Figure 2). No interaction (ATR treatment x sample) effects were found, and only sample effects were significant for horizontal activity, margin and center distance [F‘s (7, 189) = 30.903–61.165, *p* < 0.05].

### Effects of the chronic exposure in the mRNA expression of genes related to the metabolism of GABAergic and glutamatergic systems

To evaluate the effects of chronic ATR exposure on the GABAergic and glutamatergic systems, the mRNAs’ expression for several genes was assessed in the striatum, nucleus accumbens, ventral midbrain, prefrontal cortex, and hippocampus (Table 3).

**TABLE 3:** Summary of the effects of chronic ATR exposure on the expression genes associated with GABAergic and glutamatergic systems.

Real time PCR													
Brain region	Glutamatergic system								GABAergic system				
	*Gls*	*Gls2*	*Slc17a6*	*Grin1*	*Grik1*	*Gria1*	*Slc1a3*	*MGLUR1*	*GAD65*	*GAD67*	*Gabra2*	*Slc6a1*	*Slc32a1*
Striatum	↑	**-**	**-**	**-**	**-**	↑	↑	↑	**-**	↑	**-**	**-**	↑
Nucleus accumbens	↑	**-**	**-**	**-**	**-**	**-**	**-**	**-**	**-**	**-**	**-**	**-**	**-**
Ventral midbrain	**-**	**-**	**-**	**-**	**-**	**-**	**-**	**-**	**-**	**-**	**-**	**-**	↑
Prefrontal cortex	**-**	↑	**-**	**-**	**-**	↑	**-**	**-**	**-**	**-**	**-**	**-**	**-**
Hippocampus	**-**	↑	↑	↑	**-**	**-**	**-**	**-**	**-**	**-**	**-**	**-**	**-**

1 mg ATR/kg.

10 mg ATR/kg.

**FIGURE 3:**
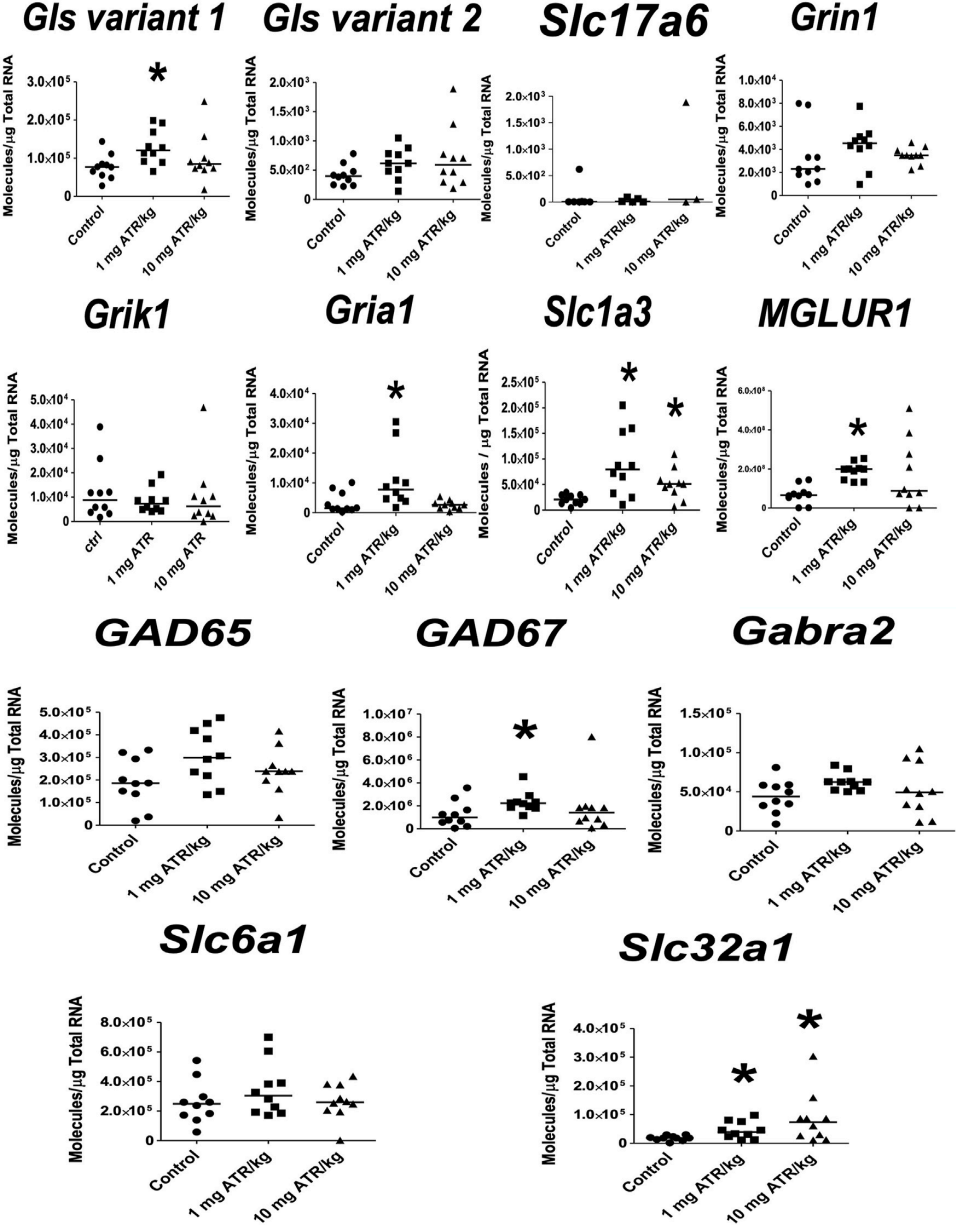
Chronic exposure to 1 or 10 mg ATR/kg increased the mRNA expression of glutaminase variant 1 (*Gls*), N-methyl-D-aspartate receptor (*Gria1*), excitatory amino acid transporter 1 (*Slc1a3*), and metabotropic glutamate receptor 1 (*MGLUR1*) of the glutamatergic system and increased the expression of glutamate decarboxylase 67 (*GAD67*) and the vesicular GABA transporter (*Slc32a1*) of the GABAergic system in the striatum. Data were analyzed by Kruskal-Wallis test for overall effects; in case of significant effects, Mann–Whitney U test was used for pairwise comparisons. * Denotes differences between 1 or 10 mg ATR/kg group and the control group, *p* < 0.05. (*n* = 10).

ATR treatment effects were found for *Gls variant 1*, *Gria1*, *Slc1a3, MGLUR1*, *GAD 67*, *Slc32a1* mRNA expression [H´s = (2, N = 30) = 7.185–10.74, *p* = 0.0254] in striatum. Post hoc analysis showed that *Gls variant 1*, *Gria1*, *Slc1a3*, *MGLUR1*, *GAD67*, and *Slc32a1* mRNA expression increased in the 1 mg ATR/kg group (U´s = 5.00–20.00, *p* < 0.05) while the mRNA expression of *Slc1a3 and Slc32a1* increased also in the 10 mg ATR/kg group (U´s = 15.00 and 18.00, *p* < 0.05) in comparison to the control group (Figure 3).

**FIGURE 4:**
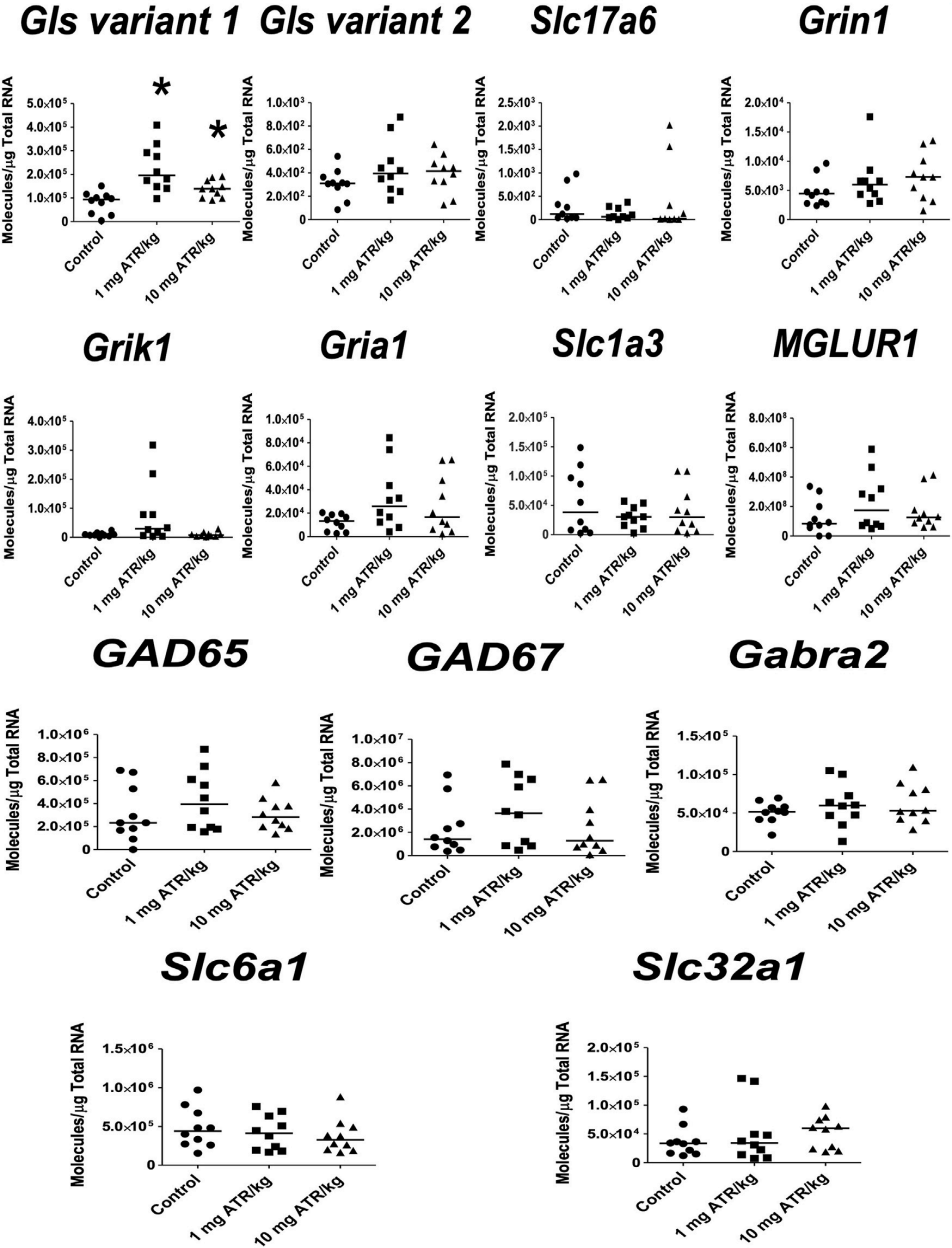
Chronic exposure to 1 or 10 mg ATR/kg only increased the mRNA expression of glutaminase variant 1 (Gls) of the glutamatergic system in the nucleus accumbens. Data were analyzed by Kruskal-Wallis test for overall effects; in case of significant effects, Mann–Whitney U test was used for pairwise comparisons. * Denotes differences between 1 or 10 mg ATR/kg group and the control group, *p* < 0.05. (*n* = 10).

In the nucleus accumbens, significant ATR treatment effect was found for *Gls variant 1 mRNA expression* [H = (2, *n* = 30) = 14.55, *p* = 0.007]. Post hoc analysis showed increases in the mRNA expression of *Gls variant 1* in the 1 mg ATR/kg group (U = 6.00, *p* = 0.0003) and the 10 mg ATR/kg group (U = 17.00, *p* < 0.0115) in comparison to the control group (Figure 4).

**FIGURE 5:**
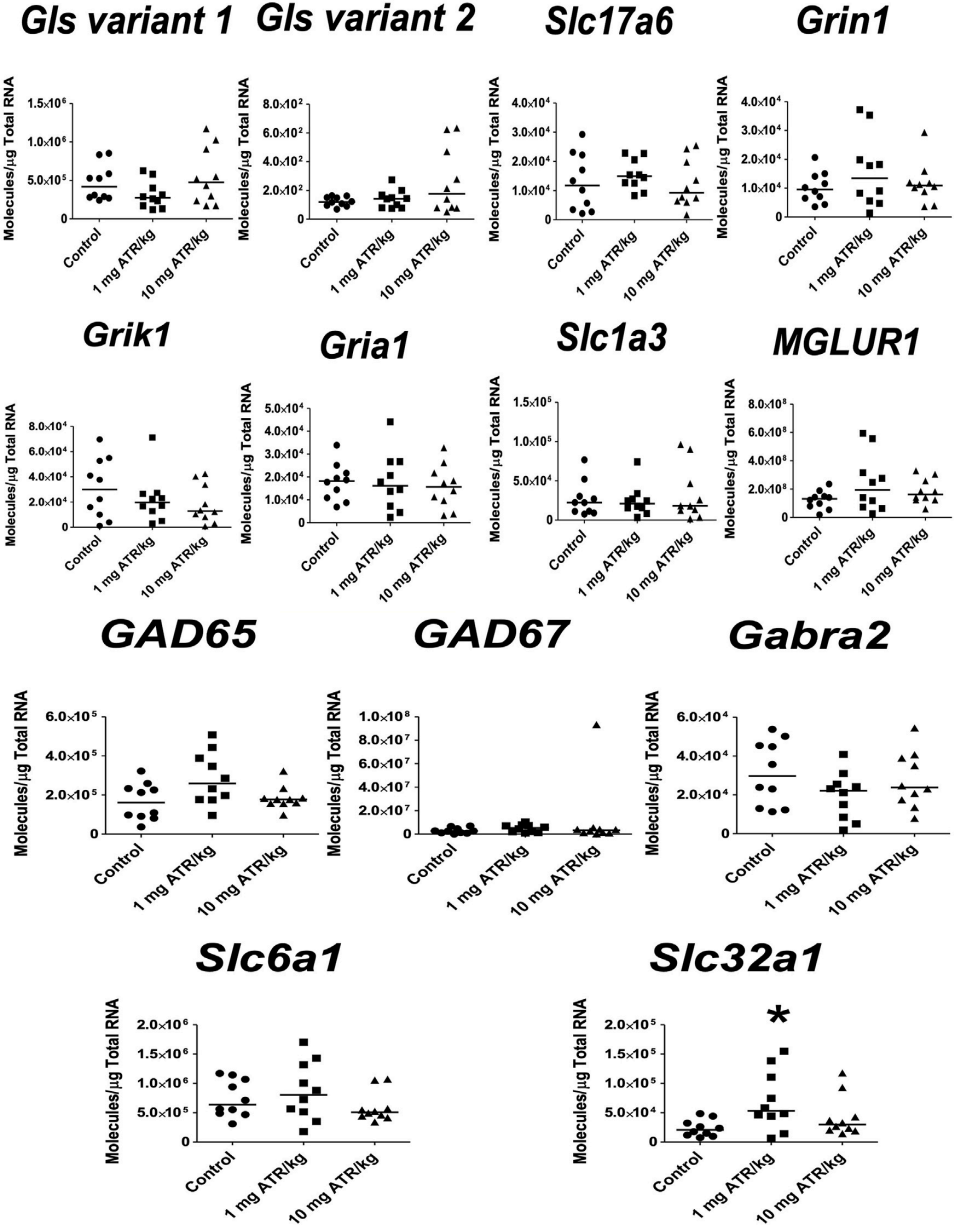
Chronic exposure to 1 mg ATR/kg increased the mRNA expression of vesicular GABA transporter (*Slc32a1*) of the GABAergic system in the ventral midbrain. Data were analyzed by Kruskal-Wallis test for overall effects; in case of significant effects, Mann–Whitney U test was used for pairwise comparisons. * Denotes differences between 1 or 10 mg ATR/kg group and the control group, *p* < 0.05. (*n* = 10).

For the ventral midbrain, only ATR treatment effects were found for *Slc32a1* mRNA expression [H = (2, *n* = 30) = 6.196, *p* = 0.0451]. Post hoc analysis showed increases in the mRNA expression of *Slc32a1* in the 1 mg ATR/kg group (U = 6.00, *p* = 0.0003) in comparison to the control group (Figure 5).

**FIGURE 6:**
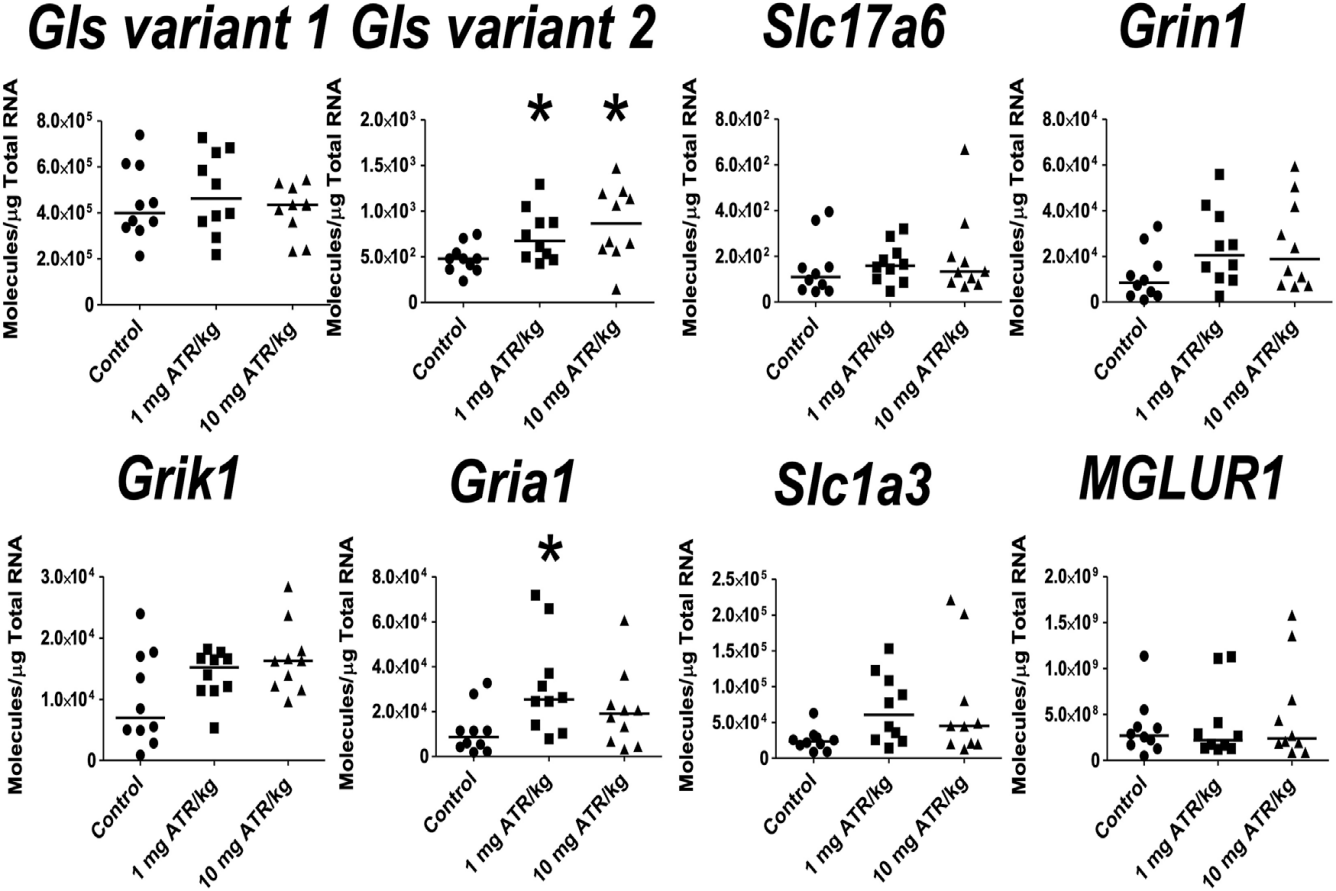
Chronic exposure to 1 or 10 mg ATR/kg increased the mRNA expression of glutaminase variant 2 (*Gls*) and α-amino-3-hydroxy 5 methyl-4-isoxazole propionic acid receptor (*Gria1*) of the glutamatergic system in the prefrontal cortex. Data were analyzed by Kruskal-Wallis test for overall effects; in case of significant effects, Mann–Whitney U test was used for pairwise comparisons. * Denotes differences between 1 or 10 mg ATR/kg group and the control group, *p* < 0.05. (*n* = 10).

ATR treatment effects were found for *Gls variant 2 and Gria1* mRNA expression [H´s = (2, *n* = 30) = 7.829 and 6.849, *p* < 0.05] in prefrontal cortex. Post hoc analysis showed that *Gls2 and Gria1* mRNA expression increased in the 1 mg ATR/kg group (U´s = 21.00 and 19.00, *p* < 0.05) while the mRNA expression of *Gls variant 2* increased also in the 10 mg ATR/kg group (U = 18.00, *p* < 0.0171) in comparison to the control group (Figure 6).

**FIGURE 7:**
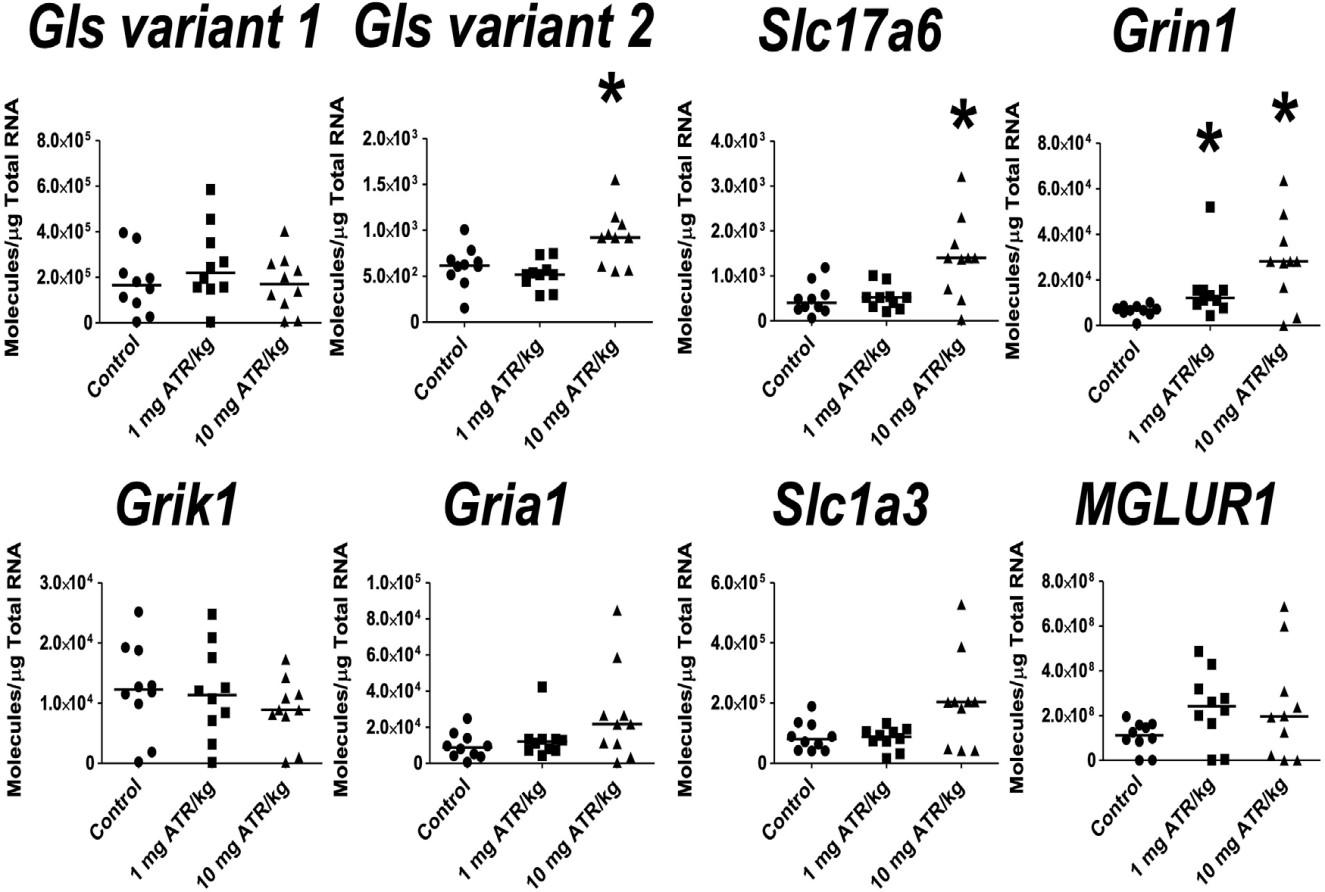
Chronic exposure to 1 or 10 mg ATR/kg increased the mRNA expression of glutaminase variant 2 (*Gls*), vesicular glutamate transporter 2 (*Slc17a6*), and N-methyl D-aspartate receptor (*Grin1*) of the glutamatergic system in the hippocampus. Data were analyzed by Kruskal-Wallis test for overall effects; in case of significant effects, Mann–Whitney U test was used for pairwise comparisons. * Denotes differences between 1 or 10 mg ATR/kg group and the control group, *p* < 0.05. (*n* = 10).

In the hippocampus, significant ATR treatment effects were found for *Gls variant 2, Slc17a6* and *Grin1* mRNA expression [H´s = (2, N = 30) = 8.422–10.54, *p* < 0.05]. Post hoc analysis showed increase in the mRNA expression of *Grin1* in the 1 mg ATR/kg group (U = 13.00, *p* = 0.0058) and increase of *Gls variant 2, Slc17a6* and *Grin1 mRNA expression* in the 10 mg ATR/kg group (U´s = 13.00–23.00, *p* < 0.05) in comparison to the control group (Figure 7).

## Discussion

Since 2011 we have been interested in exploring the effect of atrazine on neurotransmitters; we have shown that depending on the dose and length of exposure, there are changes in behavior, activation of brain regions (using the c-Fos expression), and changes in monoamines, GABA, glutamate and glutamine (Bardullas et al., 2011; Bardullas et al., 2013; Rodriguez et al., 2013; Marquez-Ramos et al., 2017; Rodriguez et al., 2017; Chavez-Pichardo et al., 2020). This paper explores the changes at the mRNA levels of several genes involved in the production, vesiculation, reuptake, and receptors of GABA and Glu in the brain of rats chronically exposed to the widely used herbicide atrazine.

Chronic ATR exposure to 1 or 10 mg ATR/kg increased the mRNA expression of some genes of the glutamatergic and GABAergic systems involved in the production, vesiculation, reuptake, and some of its receptors in the prefrontal cortex, nucleus accumbens, striatum, hippocampus, and ventral midbrain. The increased expression of genes related to GABAergic and glutamatergic systems could be related to the hyperactivity found in rats exposed to 10 mg ATR/kg and already reported in this chronic paradigm exposure of ATR (Bardullas et al., 2011; Chavez-Pichardo et al., 2020). These results support our previous study that showed the GABAergic and glutamatergic systems as targets of the chronic ATR exposure and the susceptibility not only of the basal ganglia but other structures such as the hippocampus and the prefrontal cortex.

The striatum is the brain region that presents more changes due to chronic ATR exposure in the mRNA expression of GABAergic and glutamatergic systems. This brain region is composed mainly of GABAergic medium spiny neurons which receive glutamatergic input predominantly from the cortex and the thalamus, and they send GABAergic output to the entopeduncular nucleus (EP), or internal segment of the globus pallidus in primates (Gpi) and/or substantia nigra pars reticulata (SNpr) (Bolam et al., 2000). In the striatum, 1 mg ATR treatment increased the mRNA expression of glutaminase transcript variant 1 (*Gls*; enzyme present in glutamatergic and GABAergic neurons and astrocytes) (Cardona et al., 2015), its function is to convert glutamine into glutamate and ammonium ions, possible implying the increased production of glutamate in this brain region, which is used as GABA precursor by medium spiny interneurons. The increased mRNA expression of Gls could be associated with a higher demand in glutamate production, which could be associated with the decreased levels of glutamate in the group exposed to 1 mg ATR/kg previously reported by Chavez-Pichardo et al., 2020. In regard to the reuptake of glutamate, we found increased the mRNA expression of the gene of the excitatory amino acid transporter (Slc1a3) (1 and 10 mg ATR), also known as GLAST, present in the glutamatergic neurons, which transports glutamate inside the cell implying the increased reuptake of glutamate by glutamatergic terminals. These results support those from a previous study that reports alterations in glutamate tissue levels and the increased release of basal or evoked glutamate with high potassium in STR (Chavez-Pichardo et al., 2020). Furthermore, there were also increased mRNA expression of subunit 1 of the ionotropic glutamate receptor AMPA (Gria1) (1 mg ATR group) and the glutamate metabotropic receptor 1 (MGLUR1) (1 mg ATR group), which indicates modifications in the fast, as well as slow responses to glutamate in this brain region. Moreover, for the GABAergic system in the striatum, there was increased mRNA expression of the main active enzyme in the brain related to GABA production (GAD67), which catalyzes the decarboxylation of glutamate to GABA in the group chronically exposed to 1 mg ATR. In this respect, GAD67 expression could reflect the increased synthesis of interneuronal GABA within the striatum (Lau and Murthy 2012). Also, increased mRNA expression of the solute carrier family 23 member 1 (Slc32a1) was found, a protein related to vesiculation of GABA in both groups exposed to ATR, implying the increased vesiculation of GABA in the striatum. These alterations in the mRNA expression of these genes could be related to the neurochemical disruptions already reported by Chavez-Pichardo et al. (2020). The increased expression of genes associated with the reuptake of glutamate (Slc1a3), vesiculation of GABA (Slcl32a1), and glutamate receptors (Gria1 and MGLUR1) in both the glutamatergic and GABAergic systems show the susceptibility of these neurotransmitter systems to this herbicide. It is important to note that ATR has been reported to alter striatal dopamine production, reuptake, and vesiculation. In this respect, Hossain and Filipov (2008) found a dose-dependent (1–250 uM) decrease in DA vesiculation using a preparation of synaptic vesicles from striata of Sprague-Dawley rat. These data show that not only the dopaminergic system is affected after exposure to ATR (Coban and Filipov 2007; Bardullas et al., 2011; Bardullas et al., 2013; Lin et al., 2013; Rodriguez et al., 2013; Marquez-Ramos et al., 2017), but that several proteins related to the metabolism of GABAergic and glutamatergic systems are targets of this herbicide.

The NAcc or ventral striatum is a brain region like the striatum composed of GABAergic neurons that receive glutamatergic input from the amygdala, hippocampus, prefrontal cortex, and dopaminergic input from the ventral tegmental area. The NAcc sends GABAergic projections to globus pallidus, pedunculopontine tegmentum, and substantia nigra. It has been assumed that NAcc is a limbic–motor interface that incorporates context information and drives reward to influence motivated behavior (Mogenson et al., 1980). In this region, the chronic exposure to 1 or 10 mg ATR increased mRNA expression of the Gls variant 1, which could be associated with a higher demand for glutamate to produce GABA. A previous study reported increased GABA levels in nucleus accumbens in the group exposed to 10 mg ATR (Chavez-Pichardo et al., 2020). Thus, the increased levels of GABA in NAcc could be contributing to hyperactivity presented by the 10 mg ATR/kg group in this and a previous report (Chavez-Pichardo et al., 2020).

The ventral midbrain is composed of GABAergic, glutamatergic, dopaminergic cells, among other cell types. In this brain region, we found increased mRNA expression of the solute carrier family 23 member 1 (Slc32a1), a protein related to vesiculation of GABA in both groups exposed ATR, possibly implying the increased vesiculation of GABA in the ventral midbrain. This result could be associated with the increased content of GABA in this brain region of rats exposed to 10 mg ATR as reported in a previous study by our group (Chavez-Pichardo et al., 2020). Furthermore, Rodriguez et al. (2013) reported this brain region as a target of ATR exposure when they reported decreased mRNA expression of dopamine transporter (DAT) and tyrosine hydroxylase (th) and increased mRNA expression of the vesicular monoamine transporter-2 (vmat-2) in substantia nigra of Sprague-Dawley rats repeatedly exposed to 100 mg ATR. Together these data could indicate alterations at the vesiculation stage of dopamine and GABA in the ventral midbrain.

The prefrontal cortex has excitatory (glutamatergic) neurons and inhibitory (GABAergic) interneurons expressing several peptides (Szilagyi et al., 2011). PFC plays a vital role in executive functions (goal-directed behavior) such as the decision to execute actions in working memory, depression, among others (Warden et al., 2012). The increased mRNA expression of glutaminase transcript variant 2, also known as Gls-encoded alternative-spliced short transcript glutaminase variant or GAC (Cardona et al., 2015), could be associated with the decreases in glutamine levels found in the prefrontal cortex of rats chronically treated with 1 or 10 mg ATR for 14 months (Chavez-Pichardo et al., 2020). Also, we found increased mRNA expression of subunit 1 of the ionotropic glutamate receptor AMPA (Gria1) (1 mg ATR group), which could indicate alterations in the fast response to glutamate in this brain region. These alterations could be associated with a report by Bardullas et al. (2011), who in rats chronically treated with 10 mg ATR/kg found disruptions on behavioral tasks such as the spontaneous alternation task and in the non-delayed random foraging paradigm, these behavioral tasks are strongly dependent on the integrity of the PFC (Robbins 2005).

Hippocampus is related to learning and memory formation. Like the cortex, the hippocampus is composed of pyramidal cells (glutamatergic cells) and interneurons (GABAergic cells) (Szilagyi et al., 2011). And similar to the prefrontal cortex, chronic ATR exposure to 10 mg ATR/kg increased mRNA expression of glutaminase transcript variant 2, which could be associated with glutamine metabolism alterations. However, there are no reports regarding the effects of ATR exposure on GABA or glutamate metabolism in the literature for comparison. Also, the increased mRNA expression of Slc17a6 implies possible alterations in glutamate vesiculation and consequent glutamate release. Regarding the effects of ATR exposure on glutamate receptors, the increased mRNA expression of glutamate ionotropic receptor NMDA type subunit 1 (Grin1) could indicate modifications in the fast response to glutamate in both groups exposed to ATR. In this respect, some studies have reported increased oxidative stress on this brain region after the oral exposure to 200 mg/kg body weight/day for 30 successive days (Ahmed et al., 2022). Also, maternal or developmental ATR exposure impairs spatial learning and memory, damages hippocampal morphology, and reduces expression of genes and proteins associated with mechanisms of learning and memory (Li et al., 2018; Wang et al., 2019).

Interestingly, in some cases, the 1 mg ATR/kg group but not the 10 mg ATR/kg group showed increases the mRNA expression of particular genes. This finding indicates the possibility of a non-linear relationship between ATR dose and mRNA expression response, a type of response, that is, commonly observed after the exposure to other environmental toxicants, such as lead and arsenic, as well as to endocrine disruptors (Calabrese & Baldwin, 2002; Hirsch et al., 2003; Yang et al., 2007; Lagarde et al., 2015). However, more information is needed regarding the effects of chronic exposure to small doses of ATR to clarify this observation.

These data support the notion that chronic ATR exposure produces alterations in the GABAergic and glutamatergic systems and suggests possible molecular targets in PFC, STR, NAcc, ventral midbrain and the hippocampus, regions involved in movement, addiction, memory and learning, decision making, habituation, among others brain functions. However, further studies are necessary to unravel the risk of exposure to the herbicide atrazine and its association to neurodegenerative disorders.

## Data Availability

The original contributions presented in the study are included in the article/supplementary materials, further inquiries can be directed to the corresponding author.
